# Postmortem Micro-CT of Human Fetal Heart—A Systematic Literature Review

**DOI:** 10.3390/jcm10204726

**Published:** 2021-10-15

**Authors:** Camilla Sandrini, Simona Boito, Claudio M. Lombardi, Sophie Lombardi

**Affiliations:** 1 Division of Cardiology, Department of Medicine, University of Verona, 37126 Verona, Italy; 2 Fetal Medicine and Surgery Service, Fondazione IRCCS Ca’ Granda, Ospedale Maggiore Policlinico, 20122 Milan, Italy; simona.boito@policlinico.mi.it; 3 Studio Diagnostico Eco, 20871 Vimercate, Italy; clombardi@mac.com; 4 Department of Diagnostic and Interventional Radiology, Fondazione IRCCS Policlinico San Matteo, 27100 Pavia, Italy; s.lombardi@smatteo.pv.it

**Keywords:** postmortem micro-CT, virtual autopsy, fetal heart, congenital heart disease

## Abstract

Micro-computed tomography (CT) is a non-invasive alternative to conventional macroscopic dissection for the evaluation of human fetal cardiac anatomy. This paper aims to systematically review the literature regarding the use of micro-CT to examine human fetal hearts, to illustrate its educational and research implications and to explain its possible directions for the future. A systematic literature review was conducted following the PRISMA statement to identify publications concerning micro-CT applications for the isolated human fetal heart. The search strategy identified nine eligible studies. Micro-CT is technically feasible for postmortem examination of the human fetal heart coming from early and late termination of pregnancy. It reaches high diagnostic accuracy, and it seems to perform better than autopsy in small samples or in the case of early termination of pregnancy. Applications derived from micro-CT allow multiple off-time evaluations and interdisciplinary comparisons for educational purposes and research perspectives in biological and bioengineering domains.

## 1. Introduction

Heart defects are one of the most frequent congenital malformations, occurring in roughly 1% of liveborn infants and a higher proportion of fetuses [1,2]. The global detection rate of congenital heart disease (CHD) in utero is about 60–85% [3,4,5,6,7,8]. Early prenatal diagnosis of CHD is currently feasible as early as the 11th–12th weeks of gestational age (GA) [9,10,11,12,13] thanks to progress in technology and the first trimester obstetric program of screening for fetal aneuploidies [14,15,16,17,18,19,20,21,22]. When heart malformations are suspected, in the case of a termination of pregnancy (TOP) or fetal demise, a postmortem study of the fetal heart is crucial for family counselling and educational and research purposes. Autopsy is still considered the gold standard technique for postmortem investigation, even if its limitations are well known. At early gestational ages, it can be technically limited and not adequately accurate. Moreover, other factors, such as its destructive nature and familial, cultural and anamnestic issues can reduce its acceptability to the parent [23,24,25,26]. Currently, there are different non-invasive alternatives to an autopsy that allow the study of the human fetal heart. Contrast-enhanced micro-computed tomography (micro-CT) is one of the alternative methods that has proven to be feasible in the postmortem evaluation of the human fetal heart since the 8° WG, reaching adequate detection rate compared to the gold standard technique [27,28,29,30,31,32,33,34]. Besides the clinical domain, it can be used for educational and teaching purposes, having the potential to generate overriding medical and non-medical areas for future scientific and engineering advancements in the field of pediatric cardiology [35,36,37,38,39,40]. When compared to other non-invasive postmortem techniques, micro-CT seems to have a better balance between advantages and limitations for studying isolated fetal hearts [41,42,43,44]. Previously published studies on this technique are heterogeneous in their objectives, studied population, methods and imaging analysis [27,28,29,30,31,32,33,34].

The purpose of this review is to systematically review the use of postmortem contrast-enhanced micro-CT to examine human fetal hearts, clarifying the possible clinical implications and its research/educational purposes.

## 2. Materials and Methods

This systematic literature review was conducted following the Preferred Reporting Items for Systematic Reviews and Meta-Analyses (PRISMA) statement. Database searches for English language studies were performed on PubMed and MeSH until May 2021, using the medical subjects “micro CT “, “microfocus computed tomography”, “postmortem micro CT”, “cardiac”, “heart”, “virtual autopsy”, in combination with the Boolean operator (AND) when appropriate. All matched papers were hand checked by two independent investigators (C.S., C.M.L.) to ensure they fulfilled the inclusion criteria. Studies were excluded if they met at least one exclusion criterion.

### 2.1. Eligibility Criteria

Studies were included if they fulfilled all the following criteria: (1) studies analyzing the human fetal heart (isolated or not); (2) clinical articles, reviews and case reports on the use of micro-CT in clinical scenarios or for research/educational purposes. A study was excluded if it met at least one exclusion criterion: (1) preclinical studies concerning animal experiments; (2) neonatal, childhood or the adult population; (3) studied organs other than the heart; (4) non-English studies.

### 2.2. Data Extraction and Tabulation

The following study characteristics were extrapolated for comparison and tabulated: number of specimens; type of specimen; incidence of heart disease; weight and dimension of specimens; GA at TOP; type of specimen conservation; type of specimen preparation for micro-CT; micro-CT technical properties for acquisition; isotropic voxel size; methods of analysis for the data coming from micro-CT; availability of comparison with autopsy.

### 2.3. Data Analysis

Meta-analysis is not feasible due to the heterogeneity in the methods of previously published studies. Therefore, descriptive analysis was used to explain the application of micro-CT in both clinical and research/educational scenarios. Studies investigating the use of micro-CT in clinical scenarios were presented according to extrapolated and tabulated data, as listed above.

## 3. Results

### 3.1. Selected Studies

**Figure 1:**
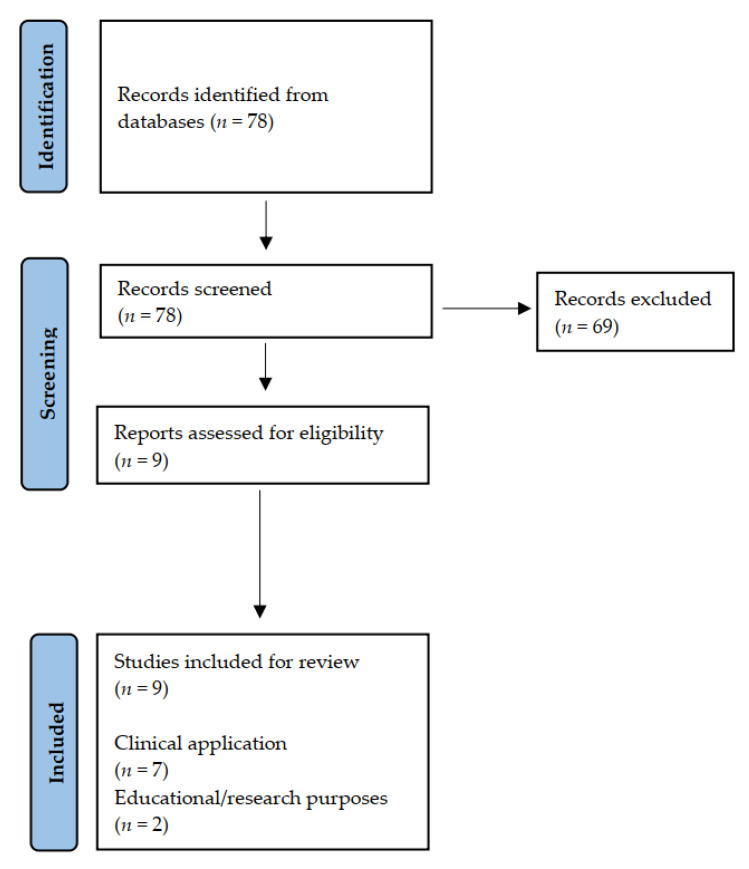
PRISMA flow chart.

The literature search yielded 78 articles that were manually screened according to inclusion and exclusion criteria, of which 69 were excluded and 9 were included for analysis. They were divided into two groups for further analysis: micro-CT in a clinical scenario (*n* = 7) and micro-CT for research/educational purposes (*n* = 2). (Figure 1).

### 3.2. Micro-CT in Clinical Scenario

A total of 7 studies belong to this subgroup (Table 1).

Specimens: population consisted of a postmortem of the whole fetal body or isolated heart (or heart and lungs in one study, in the case of prenatally suspected heterotaxy syndrome). CHD was variably represented in studies. The dimension and weight of the samples varied according to their gestational age and were not always reported in studies.

Gestational age: gestational age at TOP of studied specimens varied from 8 to 35 weeks.

**Conservation and preparation of specimens**: Specimens always needed to be fixed in a 4% PFA or 10% formalin solution to prevent tissue degradation. Staining preparation of the specimens with an iodine contrast agent (Lugol) was necessary to visualize cardiac soft tissue. It is necessary to balance the positive effect of staining preparation (the higher the contrast of the concentration, the greater difference in staining and the more uniform penetration) to the negative ones (for contrast media, the lower the isotonic to biological tissue ratio and the longer the staining time, the more tissue shrinkage and distortion). Staining preparation varied between authors and was performed by immersing the whole body, or the studied fetal organ, in the staining solution. Lombardi et described and used a protocol with three different types of staining preparation according to the weight of the samples (concentration of Lugol 25, 50, 50%; staining time 48, 48, 72 h for samples of weight <1 g, 1–2 g, >2 g, respectively) [27]. The same protocol was applied by Sandrini et al. [29]. Hutchinson et al. used a solution of 10% formalin and Lugol with a total iodine content of 63.25 mg/mL in a 1:1 ratio for over 48 h [28]. The same protocols were applied for the whole body micro-CT, except for a longer staining time (72 to >96 h) [31,32]. For the smallest samples, a 15% iodine concentration solution was chosen [30]. Little is described about the effects of contrast preparation on tissue deformities. All authors assumed that iodine preparation had some effect on tissue but that these effects did not alter the subsequent autoptic evaluation. Lupariello et al. recently demonstrated that the iodine-based staining procedure does not negatively affect cardiac samples, paraffin embedding, microtome cutting and slides preparation, nor limit the histologic and immunohistochemistry of staining due to an absence of interference with antigenic reactivity. However, they observed modifications to the cyto-architecture due to protein disarrangement, with an increase in intermediate filaments and intercellular spaces in all samples, independent of the pre-scanning contrast preparation. These observations highlight the possible role of micro-CT thermal heating in secondary and tertiary protein structures, which are influenced by heat. This could interfere with the microscopic diagnosis of some heart diseases [45]. Some authors reported the weight pre- and post-staining, showing a reduction in weight after sample preparation, which varied with the level of iodine concentration [29].

**Micro-CT technical properties**: Some specific scanner parameters are indicated by the vendors, while others depend on the type and dimension of specimens. Micro-CT resolution varied from 9 to 121.7 µm, according to the dimensions of the samples. The bigger the fetuses, the lower the image resolution, as the object was more distant from the X-ray sources. Some authors solved this problem by scanning specimens in different runs to reconstruct the whole body [31]. When focusing on single organs, scanning smaller and more homogeneous specimens enables higher resolution and decreases noise from surrounding tissue.

**Methods of analysis of micro-CT data**: Expertise in biological aspects of tissue preparation and conservation, CT execution, image optimization, heart anatomy and the anatomy of congenital heart disease, are all required to perform and interpret micro-CT correctly. It is an “interface zone” where different disciplines are required to work together. Indeed, authors of published studies come from different medical and biological domains. Moreover, as CT machines are not present in a medical context but in research institutions, close collaboration between research and clinical fields is required, and this is evident from the authors’ affiliations.

In 2014, Lombardi et al. published a pilot study on the feasibility of postmortem micro-CT for evaluating the whole body of a human fetus and ex vivo isolated hearts coming from the first and second trimester of pregnancy, comparing micro-CT (available for all cases) with conventional autopsy (available only for cases with GA > 13 weeks). Isotropic voxel size was 9 to 35 µm. Twenty-one samples were analyzed (7 whole bodies of GA 7–17 weeks, 14 isolated fetal hearts of GA 11–22 weeks, 6 prenatal diagnoses of cardiac malformation after the 15th week GA). The four-chamber view, the crux cordis, the atrioventricular valves and the three-vessel view were analyzed. Micro-CT was able to define cardiac anatomy in three cases where autopsy had failed, was concordant with conventional autopsy in six cases and added anatomic details otherwise not evaluable by autopsy in five cases. In the last group, micro-CT defined the presence of an atrioventricular septal defect in two cases and differentiated between types of heart malformations in three cases. They preliminarily demonstrated that postmortem micro-CT could be used to study the human fetal heart [27]. In 2016, Hutchinson et al. compared micro-CT to a conventional autopsy by analyzing six human fetal hearts (five CHD, one normal heart; GA between 17 and 23 weeks) with an isotropic voxel size of 19 to 31 µm. They studied the heart by defining 21 indices of cardiac anatomy. Global concordance between micro-CT and a conventional autopsy was 95.8%, sensitivity and specificity were, respectively, 85.2% and 98.9%, positive (PPV) and negative predictive values (NPV) were both 95.8%. They concluded that postmortem micro-CT provided an accurate definition of CHD and seemed to be superior to autopsy for myocardial analysis and for the evaluation of coronary arteries [28]. Following these preliminary results, Sandrini et al. compared postmortem micro-CT to conventional autopsy in 10 fetal pathological hearts (GA 12^+4^–21^+6^ weeks) by applying cardiac segmental analysis. Isotropic voxel size was 9 to 18 µm. Each sample was analyzed by both techniques by studying 25 indices of cardiac anatomy. For comparable indices (174/250, 69.6%), agreement, sensitivity and specificity were 100%. Fifty out of 76 (65.7%) non-diagnostic indices belong to the subgroup of challenging specimens (GA < 16th week and weight < 1 g), of which 84% (42/50) are evaluable by micro-CT but not by conventional autopsy (“apparent advantage of micro-CT”). Conversely, only 4% (2/50) of the indices were definable by autopsy and not by micro-CT (“missense of micro-CT”). The authors concluded that micro-CT could be a valid alternative to the conventional autopsy for the postmortem evaluation of the human fetal heart and that it may prove superior to a conventional autopsy, particularly in cases coming from early TOP or in samples with small dimensions or of low weight [29]. In 2020, Sandaite et al. demonstrated the possibility of retrieving the heart after first trimester TOP and assessed the possibility of studying the cardiac anatomy of small samples by micro-CT. Diagnostic accuracy of micro-CT in the very early scenario was out of the authors’ scope [30].

Parallel to isolated fetal heart imaging, two studies analyzed micro-CT for whole-body evaluation. In 2018, Hutchinson et al. evaluated the diagnostic accuracy of micro-CT for non-invasive whole-body human fetal autopsy in 21 early gestation fetuses (GA 11–21 weeks). Isotropic voxel size was 7.4 to 51.0 µm. Forty indices were studied (7 neurologic, 10 thoracic, 9 cardiac, 13 abdominal, 1 musculoskeletal). Overall, micro-CT agreed with autopsy findings in 35/38 diagnoses across 20 fetuses (agreement 100%, sensitivity 93.8%, specificity 100%). For comparable indices, there was a full agreement for 700/718 (97.5%), sensitivity 89.7%, specificity 99%. When analyzing first trimester fetuses (≤14 weeks GA), micro-CT analysis yielded significantly fewer non-diagnostic indices as compared to autopsy. The authors concluded that micro-CT had high concordance with conventional autopsy in early gestation fetuses (<22 weeks of GA) with a 92% diagnostic accuracy [31]. In 2020, Shelmerdine et al. analyzed 268 whole-bodied fetal specimens to describe the range of anomalies detectable on fetal micro-CT and to estimate the invasive autopsy avoidance rate. The cardiovascular system was one of the studied areas of the body. For each specimen, the micro-CT was defined as normal, abnormal or non-diagnostic. Autopsy, minimally invasive autopsy, limited autopsy or non-invasive autopsy were performed only when needed and, if performed, were used as a gold standard technique in comparison with micro-CT. The authors concluded that postmortem micro-CT could be part of a non-invasive autopsy examination [32]. Another studied body area was the brain. In 2019, Lombardi et al. demonstrated that micro-CT imaging of the early fetal brain is feasible and provides high-quality images that correlate with the histological atlas of the human brain [33].

A single paper is a case report on the utility of micro-CT in the postmortem definition of numerous small cardiac rhabdomyomas [34].

**Figure 2:**
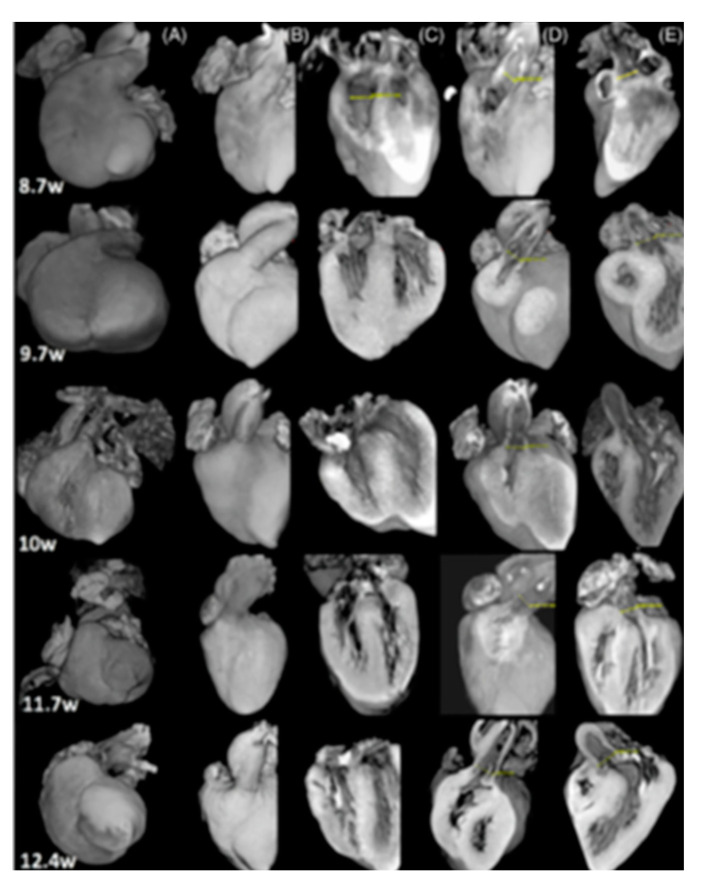
Micro-CT images of different cardiac views (columns) at different gestational ages (rows): (**A**) external apical view; (**B**) external frontal view; (**C**) four-chamber view; (**D**) right outflow view; (**E**) left outflow view [30].

**Figure 3:**
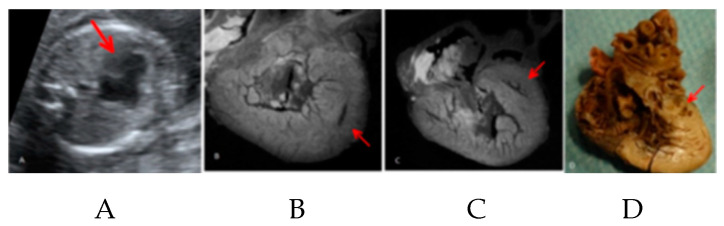
The diagnostic ability of micro-CT with prenatal fetal echocardiography and autopsy in a case of hypoplastic left heart syndrome. (**A**) Prenatal ultrasound: four chambers view shows the hypoplastic mitral valve and left heart (red arrow); (**B**) postmortem micro-CT at the level of the ventricle compares the right ventricle with the hypoplastic left ventricle (red arrow); (**C**) four-chamber view at postmortem micro-CT shows the hypoplastic left ventricle (red arrow); (**D**) four-chamber view at conventional autopsy shows the hypoplastic left ventricle (red arrow) [29].

Figure 2 shows micro-CT images of cardiac structure at different gestational ages. Figure 3 compares the diagnostic ability of micro-CT with prenatal fetal echocardiography and autopsy in a case of hypoplastic left heart syndrome.

### 3.3. Research and Educational Purposes

Micro-CT allows the acquisition of DICOM images that can be stocked and revised infinite times without the risk of wear from human manipulation or time. As DICOM images are isotropic in voxel size, they can be reconstructed in 3D datasets and navigated thanks to DICOM reader software. It makes micro-CT a suitable tool for education.

Three-dimensional printing of cardiac models from CT and MRI, and virtual reality, is an emerging field of research in pediatric cardiology for both educational purposes and clinical use in personalized pre-procedural surgical planning for complex congenital heart disease [38,39,40]. Applications of 3D printing in fetal cardiology are limited, but postmortem micro-CT seems a promising tool due to its higher resolution power and its feasibility concerning early fetuses. Shelmerdine et al. reported on the pioneering experience of 3D printing from micro-CT in human specimens for different imaging fields, including cardiology. Starting from 3D datasets, 3D-printed models can be created, allowing the true visualization of heart size and its modification at different gestational ages and used as models for cardiac morphologic courses [36]. Sandrini et al. reported experience with 3D printing from the postmortem micro-CT dataset of 21 human fetal hearts, or the heart and lungs of 12–21 weeks gestation, of which 86% suffered from congenital heart disease. They demonstrated the feasibility of 3D printing from the micro-CT dataset in the largest studied population of normal and pathological hearts, with a wide range of gestational ages. They underlined that the segmentation and printing processes are the same for normal and pathologic specimens, but reconstruction of complex anatomy may require expert guidance. Even if educational research and clinical implications were out of the aim of the study, the authors suggested that the 3D printing of fetal heart models could have a role in these scenarios [37].

## 4. Discussion and Conclusions

Postmortem micro-CT can study isolated human fetal heart, or heart and lungs, as early as the 8th week of gestation. It demonstrated the ability to define cardiac structures that cannot be visible at autopsy. Moreover, it is feasible in samples otherwise not evaluable, such as specimens coming from early TOP or very small ones. Univocal statistical interpretation of data coming from the literature regarding the diagnostic accuracy of micro-CT compared to autopsy is not possible due to small populations and to heterogeneities in methods used within published studies. We can only extrapolate that micro-CT reaches high diagnostic accuracy compared to the gold standard technique and that it seems to perform better than autopsy in samples coming from early TOP or in samples small for gestational age, of low dimension or weight. Future studies with identical methods [28,29], accurate statistical analysis and larger populations may be required to confirm this evidence. Limitations of micro-CT (i.e., cost of the equipment and of the procedure, needed expertise for sample preparation, conservation and for data acquisition and interpretation) are balanced with its advantages and the known limitations of conventional autopsies, such as parental refusal, needed pathologist’s expertise fragile samples manipulation and in the anatomy of congenital heart disease. According to existing data, we can hypothesize that micro-CT could become the initial method for the virtual dissection of the fetal heart (regardless of age at TOP, weight and dimension of the specimens and of prenatal suspicion of abnormalities). The role of the conventional autopsy could be reduced to histological examination, if needed, or for inconclusive cases. Postmortem evaluation of the fetal heart is clinically relevant for the confirmation of prenatal findings and for familial counselling. Additionally, 3D reconstruction of micro-CT datasets, and their application, allows multiple evaluations of images at different times, interdisciplinary comparison on difficult cases and for educational purposes and research perspectives in biological and bioengineering domains.

## Figures and Tables

**Table 1:** Micro-CT in clinical scenario.

Ref.	Sample (*n*.)	CHD (*n*.)	Weight (g)	Dimension (cm)	GA (w)	Fixation Protocol (Agent; Duration	Staining Protocol (Agent; Duration; %*w*/*v*)	Voxel Size (µm)	Current (µA)	Voltage (Kv)	Objective of the Study	Method	Comparison	Result
Lombardi CM 2014 [27]	21; 7 whole bodies, 14 isolated hearts	6	Whole bodies: 0.1–90 Isolate hearts: 0.1–5.2	NP	Whole bodies: 7–17 Isolate heart 11–22	4% PFA; 4–7 days	Lugol; 2–7 d; 3.75, 7.50	9, 18, 35	300, 313	80	Asses feasibility and utility of micro-CT in identifying structural anomalies	Analysis of four chamber view, crux cordis and av valves; three vessels cross sectional view of PA, Ao and SVC; no gold standard technique; descriptive analysis	Micro-CT vs. conventional dissection (available only for samples > 13 w)	Feasibility of micro-CT for identification of structural anomalies
Hutchinson JC 2016 [28]	6; isolated hearts	5	1.1–5.3	NP	17–23	10% formalin; 48 h	Lugol; 48 h; 3.75	19–31	50–135	85–125	Describe initial experience with micro-CT for the examination of complex CHD	Analysis of 21 indices of cardiac anatomy; autopsy = gold standard technique; statistical analysis	Micro-CT vs. autopsy	High accuracy of micro-CT for CHD
Hutchinson JC 2017 [34]	1; isolated heart	1	NP	NP	35	NP; NP	Iodine agent; NP; NP	NP	NP	NP	Case report	Descriptive analysis	NP	1st description of cardiac tumor identification using micro-CT
Hutchinson JC 2018 [31]	20; whole bodies	2	NP	NP	11–21	10% formalin; 72 h	Lugol; 72 h; 3.75	7.4–51	87–180	80–110	Evaluate the diagnostic accuracy of micro-CT for non-invasive human fetal autopsy for early gestation fetuses	Analysis of 9 indices of cardiac anatomy; autopsy = gold standard technique; statistical analysis	Micro-CT vs. autopsy	High accuracy of micro-CT in early gestation fetuses; better performance of micro-CT in first trimester fetuses Heart: sensitivity 90.5%, specificity 100%, PPV 100%, NPV 98.6%, concordance 98.7%
Sandrini C 2019 [29]	10; isolated hearts or heart-lungs block	10	0.39–4	0.5–1.8	12–22	10% formalin; 16–260 d	Lugol; 72 h; 3–3.75	9–18	264–500	50–89	Evaluate the diagnostic accuracy of micro-CT for diagnosis of CHD	Segmental approach; analysis of 25 indices of cardiac anatomy; autopsy = gold standard technique; statistical analysis	Micro-CT vs. autopsy	Equal diagnostic power of micro-CT as compared to autopsy for CHD; more diagnostic accuracy of micro-CT in small specimens
Shelmerdine S.C 2020 [32]	268; whole bodies	6	3–350	6–26	11–24	10% formalin; NP	Lugol; ≥96; NP	18.6–121.7	78–350	60–160	Describe the range of anomalies detectable on fetal micro-CT; estimate the invasive autopsy avoidance rate	Definition of normal, abnormal or non-diagnostic for different body areas (including cardiovascular system); autopsy/MIA/limited autopsy/NIA performed only if needed; autopsy = gold standard technique (when performed); statistical analysis (when possible)	Micro-CT vs. autopsy/limited autopsy (when performed)	Postmortem micro-CT as part of a NIA examination is feasible
Sandaite I 2020 [30]	49; isolated hearts	6	NP	0.5	8–12	10% formalin; NP	Lugol; 24 h; NP	9	NP	NP	Asses the feasibility of retrieval of heart after 1st trimester TOP; asses the feasibility of micro-CT for anatomical evaluation	Segmental approach; analysis of cardiac chambers, av connection, outflow tracts, va connection, av and semilunar valves, ventricular septum; no gold standard technique; descriptive analysis	No	Fetal heart can be retrieved after 1st trimester TOP; micro-CT can be used from as early as 8 w to describe cardiac structures

w: weeks of gestation; g: gram; cm: centimeter; NP: not provided in the text; PFA: paraformaldehyde; d: days; h: hours; av: atrioventricular; PA: pulmonary artery; Ao: aorta; SVC: superior vena cava; PPV: positive predictive value; NPV: negative predictive value; MIA: minimally invasive autopsy; NIA: non-invasive autopsy; TOP: termination of pregnancy; va: ventriculo-arterial.

## Data Availability

Not applicable.
